# Improved Skin Quality with a Novel Topical Body Serum: Distal Thighs and Back of Hands

**DOI:** 10.1111/jocd.70858

**Published:** 2026-04-14

**Authors:** Jordan V. Wang, Elizabeth T. Makino, Kuniko Kadoya, Tsing Cheng, Priscilla Huang, Roy G. Geronemus

**Affiliations:** ^1^ Laser & Skin Devon Pennsylvania USA; ^2^ Office of Roy G. Geronemus, MD New York New York USA; ^3^ Allergan Aesthetics An AbbVie Company Irvine California USA

**Keywords:** cosmeceutical, crepey, firmness, skincare

## Abstract

**Background:**

Intrinsic and extrinsic factors contribute to the loss of dermal extracellular matrix (ECM), which can result in a decline of skin quality and clinical signs of aging skin.

**Objective:**

This randomized, double‐blinded, split‐body, vehicle‐controlled, 12‐week study evaluated the efficacy of a skin body firming serum (BFS) that targets ECM renewal to improve skin quality on the distal thighs and back of hands.

**Methods:**

Adults with mild‐to‐moderate skin crepiness applied either BFS or vehicle twice daily to their assigned treatment side. Clinical investigator assessments, digital imaging, and participant self‐assessments were performed. Skin biopsies were taken from a limited number of participants for histologic analysis.

**Results:**

Participants were divided into 2 groups: treatment on the distal thighs (*N* = 11) and treatment on the back of hands (*N* = 12). Significant improvements in distal thigh firmness, texture, crepiness, and fine lines/wrinkles were observed from BFS treatment at weeks 4 and 12 over vehicle treatment and baseline. Firmness and texture on the back of hands significantly improved at weeks 8 and 12 of BFS treatment relative to baseline, with crepiness significantly improving at weeks 2 and 12. Significant improvements were seen in body skin texture (distal thighs), firmness (back of hands), and crepiness (back of hands) at week 12, and fine lines/wrinkles (distal thighs) at week 8, with vehicle treatment in comparison to baseline. Participants reported higher satisfaction with BFS than vehicle through week 12. Exploratory histologic assessments showed induction of collagen and elastic fibers.

**Conclusions:**

BFS significantly improved skin quality in both the distal thighs and back of hands, suggesting that BFS enhances body skin quality.

## 1 Introduction

As skin ages, the extracellular matrix (ECM) deteriorates and subcutaneous fat deposits‐which provide support, elasticity, and strength to the skin‐are redistributed [1]. These physiologic changes result in visible effects, including fine lines, wrinkles, skin sagging, and crepiness [2]. External factors (e.g., ultraviolet light, tobacco use, environmental toxins) also contribute to the aging appearance of the skin [3]. Although much of skincare research is focused on the face, an increasing number of studies have characterized the effects of aging on body skin, leading to regenerative treatment options [2, 4, 5, 6].

Fibroblasts are the main skin cell type responsible for dermal ECM generation (e.g., collagen proteins, elastic fibers) to provide skin support and volume. With aging and extrinsic factors, ECM protein production is slowed and ECM degradation is accelerated, resulting in loss of dermal ECM volume, integrity, and function [7]. Fibroblasts can respond to external stimulation from topical products through the application of growth factors, peptides, vitamins, and botanical extracts that can increase ECM renewal and improve clinical signs of skin aging [8, 9, 10, 11, 12].

A skin body firming serum (BFS) was shown to be tolerable and clinically effective in improving body skin quality of the upper arms and thighs [13]. BFS was developed through identifying cosmetic plant extracts and peptides that help target pathways involved in ECM renewal and body skin restoration and maintenance [14, 15]. BFS may be a treatment option for improving other body skin areas.

We evaluated the efficacy of BFS in improving skin quality on the distal thighs and back of hands. These body areas are particularly prone to developing crepiness, a wrinkled skin appearance, and loss of firmness, which BFS had previously been shown to improve [13].

## 2 Materials and Methods

### 2.1 Study Design

This randomized, double‐blind, split‐body, vehicle‐controlled, single‐center clinical trial assessed the efficacy of BFS when used over the course of 12 weeks by participants with mild‐to‐moderate crepiness on the distal thighs or back of hands. If qualified, participants were given a baseline assessment. Treatment assignment was randomized, with both participants and investigators blind to treatment. Participants were given 2 products (BFS and vehicle) and were randomly assigned to apply the active or vehicle product on their left or right side. Participants were blinded to treatment, with products pre‐labeled as “Left” or “Right” to treat either their left or right side of their distal thighs or back of hands throughout the study. Study products were applied twice daily (AM/PM), and participants were instructed to avoid washing their hands or using hand sanitizer for 1 h after application if they were treating their hands or to re‐apply both study products after washing hands (up to 5 times throughout the day). During follow‐up visits on weeks 2, 4, 8, and 12, participants completed clinical grading efficacy assessments, digital photographs, self‐assessment questionnaires, and adverse event reporting.

### 2.2 Participants

Healthy adults (aged 43–69 years) with mild‐to‐moderate crepiness (scores of 2 [mild] to 6 [moderate] on a 10‐point crepiness severity scale) on the distal thighs or back of hands were enrolled. Participants were excluded if they were pregnant, had active symptoms of any skin conditions in the test area that would interfere with the study, or had any condition according to the investigator that would make it unsafe to participate.

### 2.3 Investigator Assessments

Participants were evaluated in person during follow‐up visits using clinical grade efficacy assessments, which use a modified Griffiths 10‐point scale (0 = None, 1–3 = Mild, 4–6 = Moderate, 7–9 = Severe), on each of the participants' left and right sides of the treatment area group that they were assigned to:

For body skin firmness, scoring ranged from none (0; no sagging or droopy appearance; area appears completely smooth, firm, and taut) to severe (7–9; marked sagging and/or droopy, loose skin appearance).

For body skin texture, scoring ranged from none (0; no roughness or crepey texture; skin is completely smooth) to severe (7–9; marked roughness and/or crepey texture).

For crepiness, scoring ranged from none (0; no wrinkling, skin is flat and firm) to severe (7–9; prominent wrinkling and crinkly texture).

For fine lines/wrinkles, scoring ranged from none (0; no fine lines/wrinkles present; skin looks completely smooth and wrinkle‐free) to severe (7–9; many fine lines/wrinkles densely packed together).

### 2.4 Digital Photography

Digital photographs of representative treated areas from participants were taken using Canfield IntelliStudio with Vectra H2 System and Primos‐CR.

### 2.5 Participant Self‐Assessment Questionnaire

Participants completed 4‐point questionnaires evaluating effectiveness, overall satisfaction, and product attributes immediately after application and following continued use.

### 2.6 Histology

Skin biopsies from the distal thighs (*N* = 1) or back of hands (*N* = 2) were taken at baseline and at week 12. Samples were fixed with 10% neutral buffered formalin, embedded in paraffin blocks, and stained with hematoxylin and eosin (H&E) to visualize overall tissue structure (Epredia, Portsmouth, NH), Herovici staining for collagen fibers, and Van Gieson staining (StatLab, McKinney, TX) for elastic fibers. Images were captured with a NanoZoomer digital image scanner (Hamamatsu, Bridgewater, NJ). Comparisons were performed between BFS‐ and vehicle‐treated samples.

### 2.7 Adverse Event Reporting

Any clinical findings determined by the Investigator to be important and/or unusual were considered an adverse event (AE). Participants were asked to contact the clinic staff immediately if they experienced a reaction at any time during the study.

### 2.8 Statistical Methods

All statistical analyses were conducted on the intent‐to‐treat (ITT) population, which included all participants who received treatment and participated in at least 1 post‐baseline evaluation.

Statistical analyses were conducted using a Wilcoxon signed rank statistical test on the ITT population. Descriptive statistics were calculated for all participants who completed the baseline and any follow‐up visits. Summary statistics were conducted for all parameters collected during the study. Statistical significance was defined as *p* < 0.05 following a Wilcoxon signed rank test to compare BFS treatment versus baseline or BFS treatment versus vehicle treatment.

## 3 Results

### 3.1 Baseline Demographics

Of 23 enrolled participants, 22 (95.7%) completed the study. One participant withdrew consent and terminated the study early. Participants (96% female) self‐identified as White or Caucasian (87%), Hispanic (9%), and Black or African American (4%) (Table 1). Participants were divided into 2 groups: treatment on the distal thighs (*N* = 11) and treatment on the back of hands (*N* = 12).

**TABLE 1:** Baseline demographics.

	Distal Thigh (*n* = 11)	Back of Hands (*n* = 12)	All Participants (*N* = 23)
Sex			
Female	100%	92%	96%
Male	0%	8%	4%
Race and Ethnicity			
Black or African American	0%	8%	4%
Hispanic	8%	8%	9%
White/Caucasian	92%	84%	87%

### 3.2 Investigator Assessments

**FIGURE 1:**
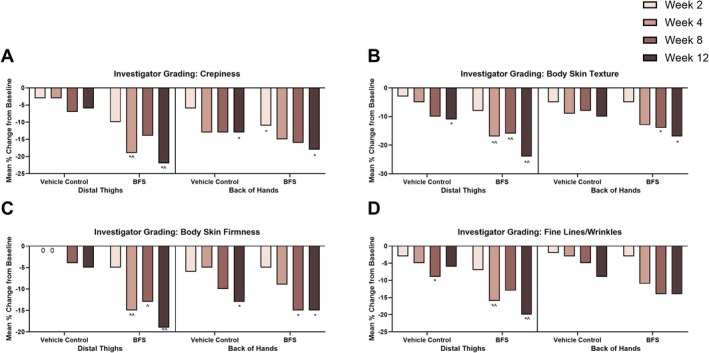
Changes from baseline in investigator‐graded skin attributes for the distal thighs and back of hands with BFS treatment. Statistically significant changes from baseline: *p < 0.05 versus baseline or ^^^ *p* < 0.05 versus vehicle.

For the distal thighs, BFS treatment led to statistically significant improvements compared with vehicle and baseline in all the assessed skin quality parameters at week 12 (Figure 1). In terms of body skin firmness on the distal thighs, BFS outperformed vehicle at weeks 4, 8, and 12 and yielded significant improvements at weeks 4 and 12 when compared with baseline. The application of BFS significantly enhanced body skin texture at weeks 4, 8, and 12 compared with both vehicle and baseline. Significant improvements in crepiness as well as fine lines/wrinkles were observed at weeks 4 and 12 when compared with both vehicle and baseline. Notably, treatment with vehicle only showed statistically significant improvements versus baseline in fine lines/wrinkles at week 8 and body skin texture at week 12.

Following use of BFS on the back of hands, significant improvements in skin firmness and texture from baseline were seen at weeks 8 and 12. Additionally, significant improvements in skin crepiness were observed at weeks 2 and 12. Use of the vehicle on the back of hands led to significant improvements in body skin firmness and crepiness in comparison to baseline at week 12. However, no statistically significant improvements in fine lines/wrinkles were seen following the use of BFS or vehicle.

### 3.3 Participant Self‐Assessment Questionnaire

**FIGURE 2:**
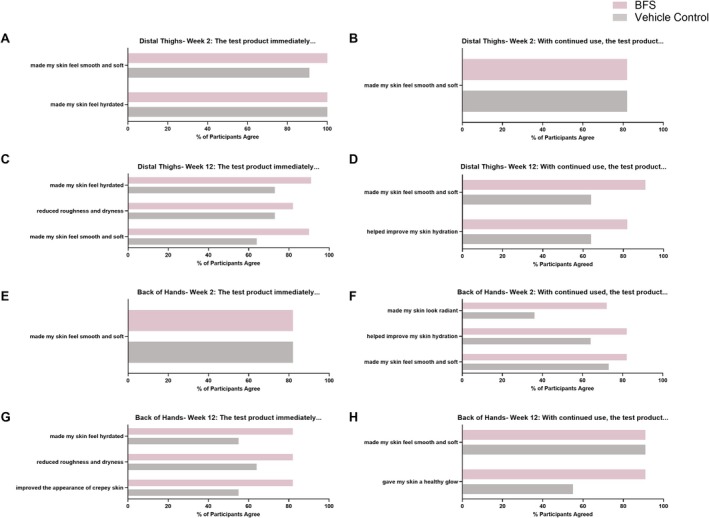
Percentage of positive responses to selected questions from self‐assessment questionnaire at weeks 2 and 12. Responses about the effects of BFS following immediate and continued use on the distal thighs (A–D) and back of hands (E–H) at weeks 2 and 12.

Most participants selected favorable responses from a series of inquiries regarding treatment efficacy immediately following and from continued use of BFS on the distal thighs (Figure 2). At week 12, most participants agreed with the statements that BFS immediately “reduced roughness and dryness” (82%), made my skin feel smooth and soft (90%), and “made my skin feel hydrated” (91%). Additionally, by week 12, the majority of participants agreed that continued use of BFS “helped improve my skin hydration” (82%) and “made my skin feel smooth and soft” (91%).

According to self‐assessment questionnaires, participants overall saw improvements in the skin on the back of their hands immediately and after continued use of BFS (Figure 2). At week 12, 82% of participants agreed that BFS immediately “improved the appearance of crepey skin,” “reduced roughness and dryness,” and “made my skin feel hydrated.” With continued use, 91% of participants agreed that BFS “gave my skin a healthy glow” and “made my skin feel smooth and soft”.

At all timepoints, at least 80% of participants also agreed with several positive statements about BFS use on both treatment areas, including that it “is convenient to use as part of my daily skin care regimen,” “spread easily in the skin,” and “absorbed easily in the skin” (Table 2). Overall satisfaction with BFS among participants was consistently high throughout the study on both the distal thighs (82% at week 12) and back of hands (91% at week 12).

**TABLE 2:** BFS attributes rated “Agree” or “Strongly Agree” by > 80% of participants at all post‐baseline visits.

BFS	
Distal Thighs	Back of Hands
Is convenient to use as part of my daily skin care regimen	Is convenient to use as part of my daily skin care regimen
Was easy to apply	Was easy to apply
Do not pill on the skin	Do not pill on the skin
Spread easily in the skin	Spread easily in the skin
Did not make my skin feel greasy	Did not make my skin feel greasy
Absorbed easily in the skin	Absorbed easily in the skin
Had a pleasant texture	Had a pleasant texture
Absorbed quickly in the skin	Absorbed quickly in the skin
Did not make my skin feel sticky or tacky	Had a neutral smell that does not bother me

### 3.4 Digital Photography

**FIGURE 3:**
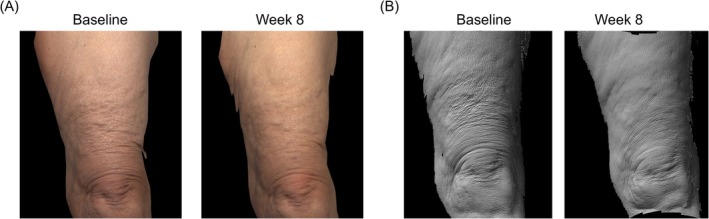
Representative photographs of the distal thighs of a White, female, 61‐year‐old participant at baseline and week 8 following BFS use in (A) standard and (B) 3D Primos images.

**FIGURE 4:**
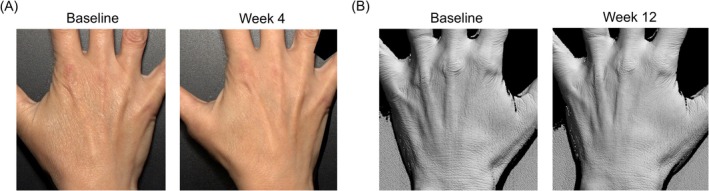
Representative photographs of the back of hands of participants following BFS use. (A) Standard images of a White, female, 57‐year‐old participant at baseline and week 4, and (B) Primos 3D images of a White, female, 58‐year‐old participant at baseline and week 12.

Photographs from representative participants showing their distal thighs and back of hands after BFS treatment are shown in Figures 3 and 4. Substantial improvements in posttreatment skin quality were observed, including reduced crepiness and improved smoothness for both treatment areas.

### 3.5 Safety

One AE, irritation of the skin, was reported on the back of the hand of the BFS side of the body. It was considered possibly related to treatment. The AE was reported on the week 8 visit.

### 3.6 Histology

**FIGURE 5:**
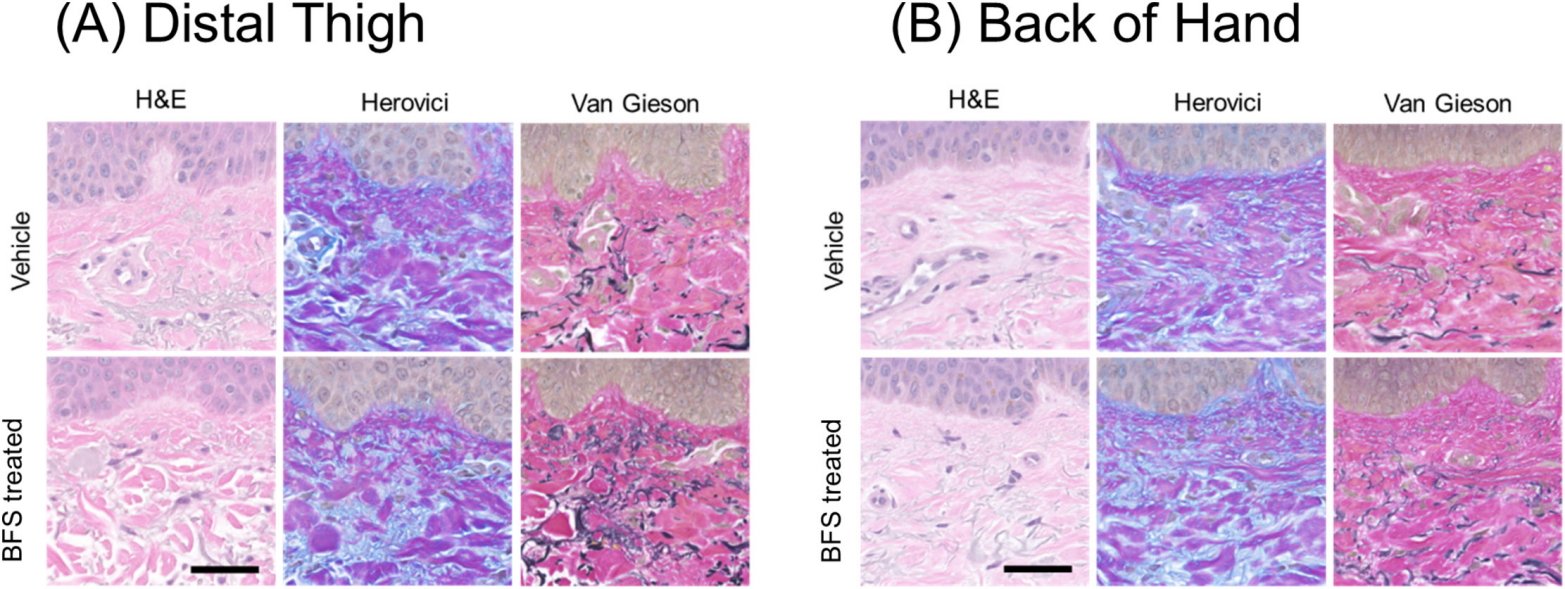
Histological assessment of biopsy samples from BFS‐treated vs. vehicle control at week 12 with H&E, Herovici, and Van Gieson staining in (A) distal thigh (56‐year‐old female participant) and (B) back of hand (63‐year‐old female participant). (Scale bars: 50 μm).

Histologic assessment of biopsies from distal thigh and back of hands was conducted by H&E staining to visualize overall structure, Herovici staining to identify newly formed collagen in blue and mature collagen in magenta, and Van Gieson staining to visualize elastic fibers in black, compared between BFS and control treated samples at week 12 (Figure 5).

Herovici staining of both distal thigh and back of hand samples of BFS treatment showed more blue‐stained fibers, indicating new/young collagen was formed compared to control. Van Gieson staining showed newly formed dermal elastic fibers in BFS‐treated tissues compared with control at week 12, especially in the upper dermis closer to the dermal‐epidermal junction (DEJ), which are known to degrade with aging. Histology also showed an increased number and more organized elastic fibers with BFS‐treated samples compared with control.

## 4 Discussion

Loss of skin firmness and increased crepiness are key body skin quality concerns for individuals, yet there are few effective treatment options readily available, especially for regular use. The distal thighs and back of hands body areas develop wrinkled and sagging skin, along with skin quality degradation, due to intrinsic and extrinsic factors [16]. Our results from a randomized, split‐body, vehicle‐controlled study showed that BFS significantly improves multiple aspects of skin quality on the distal thighs and back of hands. BFS treatment also resulted in high participant satisfaction, including with product attributes. Interestingly, our results also suggest that these body skin areas may respond differently to topical intervention. BFS treatment produced earlier improvements in the distal thighs in a subset of skin quality characteristics, while visible clinical changes on the back of hands emerged later in the treatment course. Our findings from BFS treatment therefore contribute new insights to the growing field of body skin research and underscore the importance of tailored strategies for addressing skin quality concerns in varied body areas.

Patients are often focused on the ease of product application and the rapid improvement of body skin qualities related to skin firmness, tightness, and elastic properties. To achieve these outcomes, cosmetic products may need to target biologic processes associated with ECM remodeling, cellular clearance and recycling, and lymphatic drainage [9]. BFS targets these processes via its key bioactive botanicals ingredients [15]. Preclinical data showed that BFS application stimulated the expression of collagens, elastic fiber components, as well as genes that encode proteins for autophagosome and proteasome formation, suggesting enhanced ECM remodeling [15]. These patterns of increased expression of genes related to ECM remodeling, autophagy, and proteasomal pathways were also replicated in a separate study [13]. Clinically, BFS application significantly improved skin quality in a 12‐week study treating the upper arms and thighs [13].

Combination treatment approaches for body skin quality are becoming increasingly more common, with cosmeceutical products used alongside minimally invasive procedures, such as energy‐based devices (radiofrequency, fractional lasers, cryolipolysis) to further improve outcomes. This complementary treatment strategy has been demonstrated with growth factor creams, retinoids, and antioxidants to address body skin aging and skin texture [9, 12]. Similarly, BFS can also act as a complementary treatment, as a prior study showed that its application following body contouring procedures in areas like the submental region, inner thigh, and back/bra fat enhanced skin firmness and smoothness [6]. In this previous study, participant self‐assessment questionnaires reported that BFS application enhanced the effects of the body contouring procedure. Future research should explore the potential of BFS as a complementary approach with other skin quality and body contouring procedures across body skin areas.

These new data show BFS's ability to clinically improve new body skin areas, the distal thighs and back of hands, as a standalone treatment. Although many topical skincare products are available on the market, few have demonstrated clinical efficacy in enhancing skin quality. This is particularly true for body skin, which has unique characteristics and undergoes age‐related changes that differ from facial skin [6]. As such, there is a pressing need for treatments tailored to body skin, as skin quality influences perceptions of health and overall well‐being [17, 18]. Our findings contribute valuable insights in this domain, aligning with previous research that shows how BFS treatment improves skin quality across other body areas, including the thighs, upper arms, back, and submental areas [6, 13]. This study further expands BFS's ability to treat other body skin areas, due to the different needs of skin depending on its location on the body, including skin thickness, skin laxity, and extrinsic factors like UV exposure [2]. This further underscores the importance of developing targeted skincare solutions that address the distinct physiologic traits of body skin.

Improvements in skin quality varied between the two body treatment areas, highlighting the distinct challenges of treating these areas. For the back of hands, clinical grade improvements were primarily observed following continued BFS use at weeks 8 and 12. This contrasts with other treatment areas, where improvements were noted on distal thighs as early as week 4 and on upper arms and thighs by week 2 [13]. The slower response in the hands may be attributed to their unique challenges for treatment, including high UV exposure and frequent disruption to the skin barrier from physical usage and daily hand cleansing [13]. Therefore, longer term BFS treatment may be required to achieve visible changes in hand skin quality. These slower responses may reflect the unique difficulties in achieving visible improvements in hand skin, and more broadly, how different body skin areas could respond to topical interventions. Surgical and minimally invasive hand rejuvenation procedures, such as fat grafting or injection of synthetic biomaterials, have shown improvements in aging hand appearance as early as 1 to 3 months post‐treatment [19, 20]. These procedures primarily target skin laxity and firmness, as the dermis in hands thins over time [21]. Direct comparisons between these procedural interventions with our cosmetic topical treatment, BFS, are challenging given the differences in mechanism, invasiveness, and expected magnitude of effect. Nevertheless, referencing alternative treatment modalities provides useful context for expectations around the timeline and nature of skin quality improvements in the hands.

Significant improvements following vehicle control treatment were seen from investigator‐based assessments on body skin qualities for both the back of hands and distal thighs. It is likely because it is difficult to design a true placebo for topical agents, as anything applied on the skin (i.e., lotion, oil, ointment) leads to some degree of hydration or barrier enhancement and results in visible changes to skin appearance and texture. This is a unique challenge to show greater improvements and more robust significant differences. Therefore, the magnitude and clinical relevance of the improvements observed from BFS treatment compared with vehicle treatment is critical in assessing the overall benefits to BFS. Given these challenges, the improvements observed using BFS are important achievements for addressing clinical concerns for aging hands.

Participants reported a range of benefits from using BFS, with the majority expressing agreement with positive statements about the product throughout the study. Notably, satisfaction levels were high for treatments across both body areas by week 2, with greater satisfaction for the back of hands than the distal thighs. This may be due to the high visibility of the hands, allowing participants to more readily assess improvements in skin quality. Overall, high levels of participant satisfaction with both treatment efficacy and product attributes suggest strong compliance for BFS use.

The safety profile seen here aligns with the anticipated effects of topical cosmetic products. Specifically, the low incidence of adverse events seen here, with a single case of mild irritation, is consistent with previously published reports of BFS applied to other body areas [6, 13]. Similar to this study, previously reported AEs were also mild to moderate, transient, and resolved upon discontinuation of treatment [6, 13].

Our histology from biopsied tissue suggests that BFS may promote new collagen and elastic fiber formation, both of which are critical to ECM maintenance and structure. These histological assessments were performed in only three participants; therefore, the resulting evidence should be considered preliminary and descriptive rather than confirmatory. Therefore, these results are limited to qualitative visual changes in fiber density and morphology. However, the newly formed elastic fibers seen in this study are consistent with prior ex vivo histology results, where suggesting BFS may replenish key structural ECM proteins to strengthen the dermis [7]. This includes new collagen formation (collagen type III) seen in BFS‐treated tissue compared with control, which contained older, mature collagen fibers in both studies. Larger studies employing quantitative histological analysis and a greater number of biopsy samples will be necessary to validate these initial insights.

One limitation of this study is the relatively small number of participants, which may impact the statistical power of the results. Future research should enroll additional participants and further diversify the demographics. Nonetheless, this study was designed as a clinical trial with a rigorous protocol and robust statistical analysis plan, which provides credibility to these findings. Another limitation of this study is the use of novel, non‐validated grading scales for investigator‐based assessments, which lack formal inter‐rater reliability and therefore may introduce subjectivity into outcome evaluations. However, the study was designed to address cosmetic outcomes in body areas where standardized instruments do not yet exist. This initial exploratory evaluation of BFS and treatment of understudied body skin areas will facilitate improved measurement tools and methodology in future research.

Overall, BFS provided significant improvements in skin quality in the distal thighs and back of hands as assessed by multiple investigator assessments, participant self‐assessments, and photographic observations. The majority of participants reported improved skin quality and overall satisfaction with treatment and the product. These findings support previous evidence that BFS treatment is useful for improving body skin quality and enhancing skin rejuvenation.

## Author Contributions

**Jordan V. Wang:** contributed to study execution, data collection, and interpretation. **Elizabeth T. Makino:** contributed to study design, study oversight, data analysis, and interpretation. **Kuniko Kadoya:** contributed to study design, study oversight, study execution, data analysis, and interpretation. **Tsing Cheng:** contributed to study design, study oversight, and interpretation. **Priscilla Huang:** contributed to study design, study oversight, study execution, data analysis, and interpretation. **Roy G. Geronemus:** contributed to study execution, data collection, and interpretation.

## Funding

Allergan Aesthetics, an AbbVie company, funded this study and participated in the study design, research, analysis, data collection, interpretation of data, reviewing, and approval of the publication. All authors had access to relevant data and participated in the drafting, review, and approval of this publication. No honoraria or payments were made for authorship. Medical writing support was provided by Rianne Campbell, PhD, of AbbVie Inc., and funded by AbbVie Inc.

## Disclosure

P.H., K.K., T.C., and E.T.M. are full‐time employees of AbbVie. J.V.W. and R.G.G. have nothing to disclose. ICMJE authorship criteria were met.

## Ethics Statement

The study was conducted in compliance with the Declaration of Helsinki and the standards for Good Clinical Practice. The study was reviewed and approved by Advarra IRB with participants consenting to receive study products and take part in study procedures.

## Conflicts of Interest

P. Huang, K. Kadoya, T. Cheng, and E. T. Makino are full‐time employees of AbbVie. Allergan Aesthetics, an AbbVie company, funded this study and participated in the study design, research, analysis, data collection, interpretation of data, reviewing, and approval of the publication. No honoraria or payments were made for authorship.

## Data Availability

AbbVie is committed to responsible data sharing regarding the clinical trials we sponsor. This includes access to anonymized, individual, and trial‐level data (analysis data sets), as well as other information (e.g., protocols, clinical study reports, or analysis plans), as long as the trials are not part of an ongoing or planned regulatory submission. This includes requests for clinical trial data for unlicensed products and indications. These clinical trial data can be requested by any qualified researchers who engage in rigorous, independent, scientific research, and will be provided following review and approval of a research proposal, Statistical Analysis Plan (SAP), and execution of a Data Sharing Agreement (DSA). Data requests can be submitted at any time after approval in the United States and Europe and after acceptance of this manuscript for publication. The data will be accessible for 12 months, with possible extensions considered. For more information on the process or to submit a request, visit the following link: https://vivli.org/ourmember/abbvie then select “Home”.
